# High-throughput next-generation sequencing technologies foster new cutting-edge computing techniques in bioinformatics

**DOI:** 10.1186/1471-2164-10-S1-I1

**Published:** 2009-07-07

**Authors:** Mary Qu Yang, Brian D Athey, Hamid R Arabnia, Andrew H Sung, Qingzhong Liu, Jack Y Yang, Jinghe Mao, Youping Deng

**Affiliations:** 1 National Human Genome Research Institute, National Institutes of Health (NIH), U.S. Department of Health and Human Services, Bethesda, MD 20892, USA; 2 National Center for Integrated Biomedical Informatics (NCIBI) and Center for Computational Medicine and Biology, University of Michigan, Ann Arbor, MI 48109, USA; 3 Department of Computer Science, University of Georgia, Athens, GA 30602-7404, USA; 4 Department of Computer Science, New Mexico Tech, Socorro, NM 87801, USA; 5 Harvard University, P.O. Box 400888, Cambridge, MA 02140-0888, USA; 6 Department of Biology, Tougaloo College, Tougaloo, MS 39174, USA; 7 Department of Biological Sciences, University of Southern Mississippi, Hattiesburg, MS 39406, USA

## Abstract

The advent of high-throughput next generation sequencing technologies have fostered enormous potential applications of supercomputing techniques in genome sequencing, epi-genetics, metagenomics, personalized medicine, discovery of non-coding RNAs and protein-binding sites. To this end, the 2008 International Conference on Bioinformatics and Computational Biology (Biocomp) – 2008 World Congress on Computer Science, Computer Engineering and Applied Computing (Worldcomp) was designed to promote synergistic inter/multidisciplinary research and education in response to the current research trends and advances. The conference attracted more than two thousand scientists, medical doctors, engineers, professors and students gathered at Las Vegas, Nevada, USA during July 14–17 and received great success. Supported by International Society of Intelligent Biological Medicine (ISIBM), International Journal of Computational Biology and Drug Design (IJCBDD), International Journal of Functional Informatics and Personalized Medicine (IJFIPM) and the leading research laboratories from Harvard, M.I.T., Purdue, UIUC, UCLA, Georgia Tech, UT Austin, U. of Minnesota, U. of Iowa etc, the conference received thousands of research papers. Each submitted paper was reviewed by at least three reviewers and accepted papers were required to satisfy reviewers' comments. Finally, the review board and the committee decided to select only 19 high-quality research papers for inclusion in this supplement to *BMC Genomics *based on the peer reviews only. The conference committee was very grateful for the Plenary Keynote Lectures given by: Dr. Brian D. Athey (University of Michigan Medical School), Dr. Vladimir N. Uversky (Indiana University School of Medicine), Dr. David A. Patterson (Member of United States National Academy of Sciences and National Academy of Engineering, University of California at Berkeley) and Anousheh Ansari (Prodea Systems, Space Ambassador). The theme of the conference to promote synergistic research and education has been achieved successfully.

## Introduction

With the advent of high-throughput next generation sequencing technologies, gigabases of sequence information can be obtained in just a few days. The technologies offer drastically faster and cost-effective sequence throughput and are vastly superior to shotgun sequencing due to the high volume of data and the drastically short time to sequence a whole genome or disease genome, but genome assembly is much more computational expansive. Therefore, the next generation sequencing technologies will foster enormous potential applications of supercomputing techniques in genome sequencing, epi-genetics, metagenomics, personalized medicine, discovery of non-coding RNAs and protein-binding sites. Furthermore, Next-generation sequencing will substitute microarray, the mostly used technology in genomics and bioinformatics: Like next-generation sequencing, microarrays can be used to examine thousands of genes in one experiment and can be used to obtain gene profiles, but the drawback of Microarrays are based on *hybridization*. Gene expression levels are measured by fluoresce from hybridizes but quantification of the fluorescence of vast amount of spots on a chip are often unreliable from experiment to experiment. Furthermore many DNA samples can hybridize to more than one spot, thus, generating misleading results. Next-generation sequencing overcomes problems of Microarrays by generating actual sequence reads and is ideally detect genetic mutations. To measure the gene expression level reflected by the amount of a particular RNA molecule, simply tally up the number of sequence reads corresponding to that RNA molecule rather measuring an inaccurate fluorescent spot and trying to control for all sorts of experimental variation. Gene expression can be actually more accurately obtained by counting sequence reads. Next generation sequencing has distinct advantages to obtain regulatory markings in chromatin, and to identify neural regulatory protein binds in the genome, as well as to investigate differences between stem cells and differentiated cells such as cancerous cells, and to determine how gene regulatory network can be altered by an activated external signal. Next generation sequencing is cheaper, fast, and less time consuming but computational expensive. Given situations, the development of computational techniques is important for future bioinformatics data mining. To this end, the International Society of Intelligent Biological Medicine  works with academic conferences to promote the cutting edge research.

## Research presentations

Lianjiang Wang, Jack Y. Yang and Mary Qu Yang [1] presented a novel computational approach to predict DNA-binding residues from protein sequence information using random forests. The method can be used in analysis a large amount of genomic data from high-throughput next generation sequencing and provide insight in protein-DNA interactions. Yadong Wang, Jack Y. Yang, Yunlong Liu et al [2]. developed a method for reconstructing Gene Regulatory Network using Slice Pattern Model. Such model could be applied in studying data from next generation sequencing technology. Qingzhong Liuet al [3] presented a comprehensive comparison of feature selection and classification for MALDI-MS data. Xin Wang, Yunlong Liu et al. [4] presented Genome-wide prediction of cis-acting RNA elements regulating tissue-specific pre-mRNA alternative splicing, Chacko et al. [5] presented a comprehensive splicing graph analysis of alternative splicing patterns in chicken and compared to human and mouse. Osborne, et al. [6] discussed annotations of the Human Genome with Disease Ontology. Valdimir N. Uversky, Zoran, Keith Dunker et al [6] systematically studied Unfordomics for human diseases and demonstrated links to protein intrinsic disorder with diseases. Corresponding author Uversky presented this important research and discoveries in the keynote lecture. Jianhua Ruan, Youping Deng, Weixiong Zhang [8] presented an ensemble learning approach to the problem of reverse-engineering transcriptional regulatory networks using time-series gene expression data. Hong Zhou et al. [9] studied energy profile and secondary structure that impact shRNA Efficacy. Taheri et al. [10] studied RBT-GA as a example of their novel metaheuristic for solving the multiple sequence alignment problem. Gu et al. [11] reported their studies in genomic and systems evolution in vibrionaceae species. Midic, A Keith Dunker et. al. [12] systematically studied protein disorder in the human diseasome and showed unfoldomics in human genetic diseases, Uversky delivered keynote speech summarizing their seminal research. Kim et al. [13] studied PDA as an example of automatic and comprehensive analysis program for protein-DNA complex structures. Huang et al. [14] studied differences in duplication age distributions between human GPCRs and their downstream genes from a network prospective. Zhang et al. [15] presented their research in reverse engineering module networks by PSO-RNN hybrid modeling. Jake Chen [16] talked about their Online database for comprehensive human annotated and predicted protein interactions. P. Ghosh, Joe Zhang et al. [17] formulated a computational model to predict the binding rate for the siRNA-RISC complex formation reaction. Lichtenberg et. al [18] studied promoters in human DNA repair gene pathways. Zhongxue Chen, Monnie McGee, Qingzhong Liu et al. [19] studied a distribution-free convolution model for background correction of oligonucleotide microarray data. The above 19 research papers were selected by the committee based on peer-reviews. The selected papers covered broad range of research fields; mainly include gene regulation, gene regulatory network construction, protein-DNA interaction predication and modeling, microarray data analysis, evolution study, disease study and biology database. The authors presented a variety of approaches and tools that can be used in analyzing next generation sequencing data. Meanwhile the researchers can greatly benefit from quality and quantity of data generating by next generation sequencing technology.

## Future meeting

The next annual conference will be held in same location in Las Vegas, Nevada on July 13–16, 2009. The web site, , contains further information on future meetings. The meeting is a large international conference with more than two thousand attendees from more than 80 countries annually. The conference continuously aims at promoting computational science research and education in biomedical sciences. For articles from the 2008 conference, please see , and also  for further reading.

## Competing interests

The authors declare that they have no competing interests.

## Authors' contributions

All authors provided professional services to the conferences and contributed in writing this introductory article. All authors reviewed and agreed on the content of this introductory article.
